# Supplementary figures and images for: SARS-CoV-2 disease severity and transmission efficiency is increased for airborne but not fomite exposure in Syrian hamsters
Source: bioRxiv. 2020 Dec 28:2020.12.28.424565. Preprint. [Version 1] doi: 10.1101/2020.12.28.424565 (PMC7781302; doi:10.1101/2020.12.28.424565)

**a**

14/21 DPI

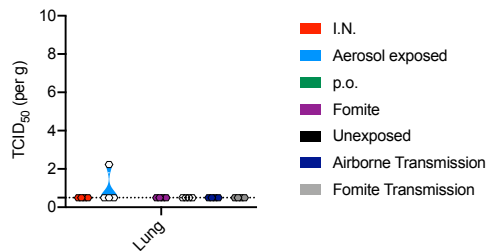**b**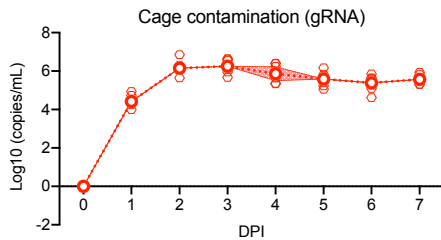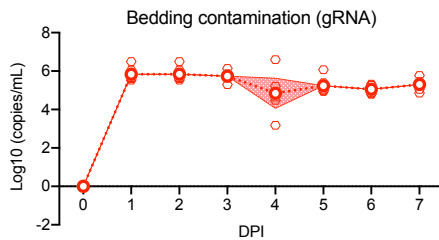**c**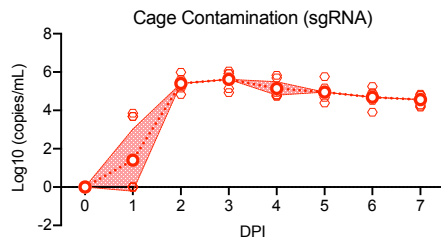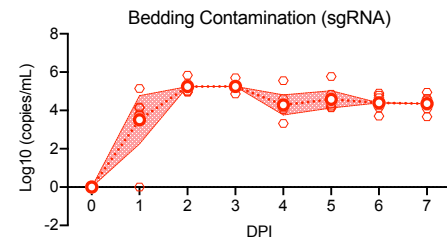

Supplement: Supplement 1 — Supplemental Figure 1: a. Violin plot of infectious SARS-CoV-2 titer in the lungs of all animals at 14 or 21 DPI. b. cage and c. bedding contamination by infected animals till 7 DPI. [file media-1.pdf]
